# Association between lactate-to-albumin ratio and mortality in hepatic failure: a retrospective cohort study

**DOI:** 10.1186/s12879-025-10783-z

**Published:** 2025-03-28

**Authors:** Huan Wu, Long Wu, Li Luo, Hai-yang Li, Bao-fang Zhang

**Affiliations:** 1 Department of Infectious Diseases, The Affiliated Hospital of Guizhou Medical University, No.28 Guiyi Street, Yunyan District, Guiyang City, Guizhou Province China; 2 Department of Anus and Intestinal Surgery, The Affiliated Hospital of Guizhou Medical University, Guiyang, Guizhou China; 3 Department of Hepatobiliary Surgery, The Affiliated Hospital of Guizhou Medical University, No.28 Guiyi Street, Yunyan District, Guiyang City, Guizhou Province China

**Keywords:** LAC, Hepatic failure, All-cause mortality, Cohort

## Abstract

**Background:**

Liver failure has a high mortality rate, and currently, there is no convenient risk predictor. The lactate-to-albumin ratio (LAR) has emerged as a promising predictor in various critical illnesses. However, its potential role in predicting all-cause mortality in patients with liver failure remains unexplored. Therefore, this study aims to investigate the correlation between LAR and all-cause mortality in patients suffering from liver failure.

**Methods:**

We retrospectively analyzed data from patients with liver failure who were admitted to the intensive care unit (ICU) between 2008 and 2019, which were gathered from the Medical Information Mart for Intensive Care IV (MIMIC-IV) database. LAR was calculated from the ratio obtained from the first measurement taken within 24 h of admission. The optimal LAR threshold was determined using the Youden index. With LAR categorized into low, middle, and high groups based on tertiles, Kaplan - Meier analysis was employed to compare mortality risks among three patient groups. Multivariate Cox proportional hazards regression models were utilized to evaluate the association between LAR and all-cause mortality in hepatic failure patients within hospital admission. Additionally, receiver operating characteristic (ROC) and smoothing curve analysis were used to assess the predictive ability, sensitivity, and specificity of LAR for all-cause mortality in patients with liver failure, and the area under the curve (AUC) was calculated. A smooth curve fitting approach and threshold effect analysis were employed to detect the potentially non-linear relationship between the LAR and the risk of all-cause mortality in patients with hepatic failure. Finally, subgroup analyses were performed to assess the relationship between LAR and prognosis across different types of liver failure.

**Results:**

A total of 902 patients with hepatic failure were included in this study. They were divided into survivors group (611 patients) and non-survivors group (291 patients) according to whether they survived during hospitalization, and the mortality rate of patients was 32.26%. The Kaplan-Meier survival curves illustrating patients in hepatic failure with elevated LAR showed a significantly heightened risk of in-hospital mortality (*P* < 0.001). We identified a non-linear relationship between LAR and the risk of hospital mortality after adjusting for potential confounders and the inflection point of LAR to be 1.33. LAR was shown to be an independent predictor of all-cause mortality within hospitalization in patients with hepatic failure by multivariate COX regression analysis (HR, 1.66; 95% CI, 1.35–2.05; *P* < 0.0001). The optimal cutoff value for separating the survival and death groups according to ROC was found to be 0.97. The AUC value for LAR was 0.755 (95% CI: 0.721, 0.789), which was higher than that for arterial blood lactate (AUC = 0.725) and serum albumin (AUC = 0.680) alone. It was not inferior even when compared to MELD (AUC = 0.677).

**Conclusion:**

LAR has demonstrated good predictive value for all-cause mortality among liver failure patients in our retrospective study.

**Supplementary Information:**

The online version contains supplementary material available at 10.1186/s12879-025-10783-z.

## Background

Liver failure represents a significant medical challenge worldwide, characterized by a spectrum of clinical manifestations ranging from liver dysfunction to severe multi-organ failure. Despite the progress made in critical care, artificial liver support system, and liver transplantation, the mortality rates among patients with liver failure still remain relatively high [1]. Early recognition and appropriate management of liver failure are crucial for preventing associated mortality. Thus, prognostic markers that accurately predict outcomes in this population are crucial for improving patient outcomes, optimizing patient management and resource allocation. In recent years, there has been a growing interest in assessing the performance and utility of existing prognostic scoring models in liver failure. Current scoring systems designed to evaluate the severity of liver failure encompass a range of models such as Child-Turcotte-Pugh (CTP), APASL ACLF Research Consortium (AARC), Model for End-stage Liver Disease (MELD), Model to Estimate Survival in Overt Hepatic Failure (MESO), Age-Bilirubin-INR-Creatinine (ABIC), Maddrey Discriminant Function (MDF), Albumin-Bilirubin (ALBI), Acute Physiology and Chronic Health Evaluation (APACHE), Chronic Liver Failure Consortium (CLIF-C), among others [2–6]. However, these models integrate various clinical, laboratory, and imaging parameters to gauge disease severity. These scoring systems necessitate the collection of multiple indicators to comprehensively assess a patient’s condition, which may inadvertently delay treatment initiation for some patients, increasing the risk of adverse outcomes. Therefore, there is a pressing need for a simple, cost-effective, and highly sensitive indicator to gauge liver failure severity, forecast short-term and long-term outcomes of disease.

Lactate, a marker of tissue hypoperfusion and metabolic stress, has garnered attention for its potential role in predicting disease severity. Among the various physiological disturbances observed in liver failure, elevated lactate levels, or hyperlactatemia, are frequently encountered [7–9]. Lactate clearance is an independent predictor of death in liver failure patients [10]. However, systemic factors such as sepsis, inflammation, and medications can contribute to hyperlactatemia by altering cellular metabolism and increasing lactate production. Thus, relying solely on lactate levels for prognostication in liver failure may overlook critical aspects of the disease process, potentially leading to delayed interventions or inappropriate management strategies. Meanwhile, serum albumin plays a crucial role in maintaining oncotic pressure, transporting substances, and modulating immune responses. One of the hallmarks of liver failure is hypoalbuminemia, which primarily reflects impaired hepatic synthetic function [11, 12]. Furthermore, hypoalbuminemia can be influenced by factors unrelated to liver function, such as nutritional status, inflammation, and renal dysfunction. Therefore, hypoalbuminemia alone cannot adequately capture the multifaceted nature of liver failure or reliably predict disease severity, which may overlook critical aspects of the disease process and lead to incomplete risk stratification and management decisions.

The LAR reflects the balance between lactate, a marker of tissue hypoperfusion and metabolic stress, and albumin, a surrogate marker of liver synthetic function and nutritional status. Several studies have evaluated that an increased LAR has been linked to worse outcomes in patients with a range of critical conditions, including acute pancreatitis, sepsis, brain injury, cardiac arrest, acute myocardial infarction, heart failure, and acute respiratory failure [13–17]. However, to the best of our knowledge, there is currently no research exploring the clinical association between the LAR and mortality among liver failure patients. The present study aims to investigate the association between LAR levels and all-cause mortality in patients with liver failure admitted to the ICU, which leveraging data from the MIMIC-IV database. This retrospective cohort study seeks to address this gap in knowledge, holds significant potential to contribute to the understanding of prognostic factors in liver failure and may pave the way for the development of improved predictive models and therapeutic strategies in managing this complex condition.

## Methods

### Database source

Data were extracted from the MIMIC-IV (version 2.2), a large, publicly accessible database developed and managed by the MIT Computational Physiology Laboratory (https://physionet.org/content/mimiciv/2.2/) [18, 19]. This database encompasses information on over 52,000 individuals admitted to the Beth Israel Deaconess Medical Center between 2008 and 2019, including each patient’s length of stay, laboratory tests, medication treatment, vital signs, and other comprehensive data. Access to this database required the successful completion of the protecting human research participants training course, the collaborative institutional training initiative course, as well as passing both the “Conflicts of Interest” and “Data or Specimens Only Research” exams (certificate number: 61863903). Following these requirements, the research team was deemed qualified to utilize the database and extract data. Importantly, to safeguard patient privacy, all patient-related information within the database is anonymized, with random codes replacing patient identification. Consequently, informed consent and ethical approval from patients were unnecessary.

### Study population

**Fig. 1:**
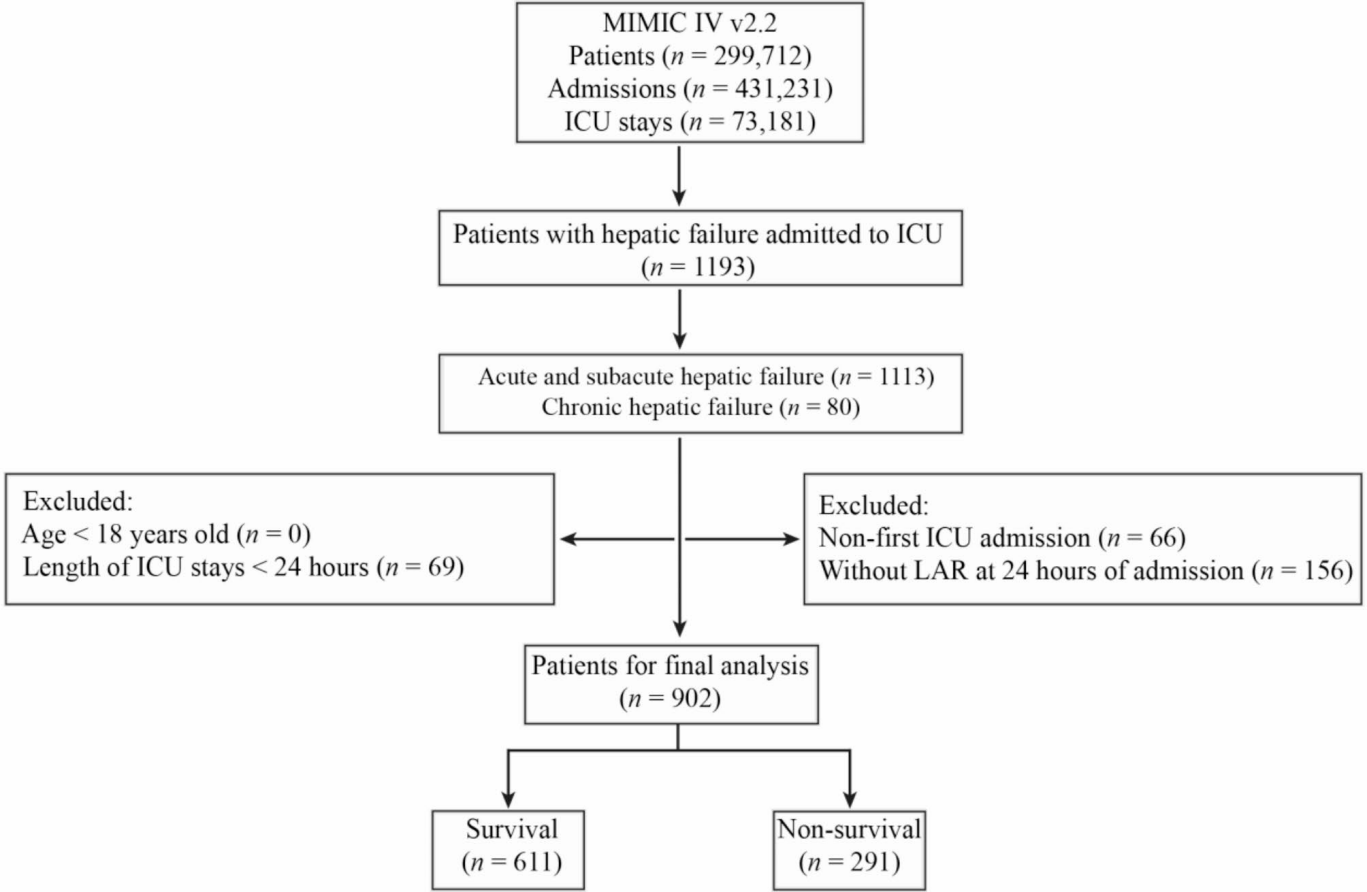
Schematic diagram of study population selection steps

This study centered on patients admitted to the ICU with hepatic failure. Intensive care patients with hepatic failure in the MIMIC-IV database included in this study were diagnosed with acute and subacute hepatic failure (K720, K7200, and K7201) and chronic hepatic failure (K721, K7210, and K7211) based on International Classification of Diseases, 10th Revision (ICD-10) codes. It’s worth noting that in the MIMIC database, the diagnosis of hepatic failure is recorded as “hepatic failure” rather than “liver failure”. Following further screening, patients who met the following criteria were excluded: (1) patients younger than 18 years of age at the time of the first admission; (2) patients who stayed in the ICU for less than 24 h; (3) lack of lactate and serum albumin measurements within 24 h of admission. Ultimately, 902 patients were enrolled in this study (Fig. 1).

### Data extraction

The data extraction process utilized PostgreSQL software (version 1.19.10821.0) and Navicat Premium software (version 15.0.29) to extract data via a running structured query language (SQL). Extraction of potential confounders encompassed demographics (age, gender, race, weight, and height), vital signs [heart rate (HR), systolic blood pressure (SBP), diastolic blood pressure (DBP), mean blood pressure (MBP), respiration rate (RR), temperature (T), and oxygen saturation (SPO_2_)], laboratory indicators [red blood cell (RBC), white blood cell (WBC), platelet (PLT), mean corpuscular hemoglobin (MCH), hematocrit (HCT), mean corpuscular volume (MCV), mean corpuscular hemoglobin concentration (MCHC), hemoglobin (Hb), red blood cell distribution width (RDW), albumin (ALB), lactate (LAC), total bilirubin (TBIL), serum glucose (GLU), direct bilirubin (DBIL), indirect bilirubin (IBIL), aspartate aminotransferase (AST), alanine aminotransferase (ALT), alkaline phosphatase (ALP), prothrombin time (PT), international normalized ratio (INR), lactate dehydrogenase (LDH), creatine kinase (CK), creatine kinase isoenzymes (CK-MB)], and severity scores [model for end-stage liver disease (MELD) and sequential organ failure assessment (SOFA)]. The LAR was calculated from laboratory data. All code utilized for the calculation of demographic characteristics, laboratory indicators, and severity scores was obtained from the GitHub website. The selection of these covariates was primarily based on our clinical experience as well as literature [13].

### Primary outcome

In this study, patient subgroups were categorized into an admission survival group (*n* = 611) and an admission death group (*n* = 291). The primary outcome of this study was all-cause mortality during hospitalization, defined as death occurring within the hospital. This encompassed deaths both in the ICU and general ward. The LAR was used as the exposure variable. Importantly, there were no patients lost to follow-up during the study period.

### Statistical analysis

In the description of population characteristics, continuous variables were presented as means ± standard deviations, while categorical variables were expressed as *n* (%). In cases where continuous variables did not follow a normal distribution, medians and inter quartile ranges (IQR) were provided. Baseline characteristics were compared using T-tests or One-Way ANOVA for continuous variables, and Pearson’s chi-squared test or Fisher’s test for categorical variables. Potential risk factors were identified through One-Way Cox Regression analysis, with variables having *P*-values less than 0.05 included in Multi-Way Cox Regression analysis to identify independent risk factors for in-hospital mortality, expressed as hazard ratio (HR) and 95% confidence interval (CI). Survival outcomes among LAR groups were compared using Kaplan-Meier (K-M) survival analysis. ROC analysis assessed the predictive ability of LAR, lactate, albumin, and MELD for mortality at admission, determining sensitivity, specificity, and AUC. The optimal cut-off value of LAR was determined using the Youden’s index, dividing LAR into high, middle, and low value groups by tertiles. Furthermore, the analysis employed smooth curve fittings and threshold effect analysis to investigate non-linear relationships. Specifically, a two-piecewise linear regression model incorporating a smoothing function was utilized to assess whether LAR had any threshold effect on the risk of hepatic failure. This involved a trial-and-error approach where turning points were selected within a predetermined interval. The turning point that yielded the highest likelihood for the model was then identified. All analyses were performed using Empower Stats software (version 4.2) and the statistical package R (version 4.3.2), with statistical significance defined as a two-sided *P* value of 0.05.

## Results

### Comparison of baseline characteristics between two groups of patients with liver failure

This study ultimately included 902 patients of hepatic failure, and the flowchart of the patient inclusion process is illustrated in Fig. 1. The baseline characteristics of the admission survival and non-survival groups are listed in Table 1. That includes 360 (39.91%) females and 542 (60.09%) males, and their average age was 60.63 years. The mortality rate of the included patients with hepatic failure was 32.26%. We noticed that hepatic failure non-survivors are older and heavier, and had lower T, SPO_2_, SBP, DBP, and MBP, while higher SOFA and MELD scores when compared to the survivors group (*P* < 0.05). The HR, RR, lactate, WBC, MCH, MCHC, MCV, ALT, AST, TBIL, DBIL, ALP, GLU, PT, INR, CK, CK-MB, and LDH in the non-survivors group were significantly higher than those in the survival group, while ALB and PLT were significantly lower, all of which demonstrated statistical significance (*P* < 0.05).

**Table 1:** Baseline characteristics between survivors and non-survivors

Variables	All-cause mortality		*P*-value
	Survivors (*n* = 611)	Non-survivors (*n* = 291)	
Gender, *n* (%)			0.319
Female	237 (38.79%)	123 (42.27%)	
Male	374 (61.21%)	168 (57.73%)	
Age, mean ± SD, year	59.24 ± 15.72	62.01 ± 15.50	0.013
Height, mean ± SD, cm	169.27 ± 10.53	169.29 ± 10.35	0.976
Weight, mean ± SD, kg	84.13 ± 22.75	87.52 ± 25.44	0.046
MELD, mean ± SD	21.12 ± 8.97	27.25 ± 9.73	< 0.001
SOFA, mean ± SD	9.01 ± 4.12	11.64 ± 4.13	< 0.001
LAR, mean ± SD	1.00 ± 1.41	2.66 ± 3.43	< 0.001
SBP, mean ± SD, mmHg	112.97 ± 14.43	106.75 ± 14.75	< 0.001
DBP, mean ± SD, mmHg	63.27 ± 11.00	59.92 ± 11.06	< 0.001
MBP, mean ± SD, mmHg	77.33 ± 10.62	74.14 ± 10.10	< 0.001
RR, mean ± SD, breath/min	19.91 ± 4.29	22.63 ± 4.92	< 0.001
HR, mean ± SD, beats/min	87.66 ± 17.08	93.90 ± 18.12	< 0.001
T, mean ± SD, ℃	36.87 ± 0.59	36.53 ± 1.04	< 0.001
SPO_2_, mean ± SD, %	96.86 ± 2.17	95.27 ± 4.98	< 0.001
WBC, median (min-max), 10^9^/L	8.20 (0.50–72.30)	12.40 (0.90–77.90)	< 0.001
RBC, mean ± SD, 10^12^/L	3.35 ± 0.79	3.26 ± 0.85	0.137
HCT, mean ± SD, %	30.66 ± 6.67	30.39 ± 7.32	0.592
Hb, mean ± SD, mg/dL	9.93 ± 2.24	9.96 ± 2.40	0.860
MCH, mean ± SD, pg	29.90 ± 3.08	30.81 ± 3.02	< 0.001
MCHC, mean ± SD, g/L	32.37 ± 1.60	32.72 ± 1.61	0.003
MCV, mean ± SD, fl.	92.37 ± 8.03	94.27 ± 8.73	0.002
RDW, mean ± SD, %	16.77 ± 3.27	17.19 ± 3.60	0.083
PLT, median (min-max), 10^9^/L	155.00 (15.00-773.00)	126.00 (12.00-540.00)	< 0.001
ALT, median (min-max), U/L	51.00 (6.00-6623.00)	134.50 (5.00-8648.00)	< 0.001
AST, median (min-max), U/L	60.00 (5.00-15960.00)	212.50 (13.00-42606.00)	< 0.001
ALP, median (min-max), U/L	115.00 (28.00-1199.00)	126.00 (22.00-2686.00)	0.030
TBIL, median (min-max), mg/dL	1.00 (0.20–45.80)	2.20 (0.20–52.20)	< 0.001
DBIL, median (min-max), mg/dL	1.50 (0.10–24.00)	5.10 (0.40–21.60)	0.011
IBIL, median (min-max), mg/dL	1.20 (0.20–11.30)	1.95 (0.30–11.10)	0.105
PT, median (min-max), s	15.55 (9.40–150.00)	17.90 (10.40-105.70)	< 0.001
INR, median (min-max)	1.40 (0.90–15.70)	1.70 (1.00-9.90)	< 0.001
Lactate, median (min-max), mmol/L	2.00 (0.50–89.00)	4.20 (0.70–23.00)	< 0.001
Albumin, mean ± SD, g/mL	3.39 ± 0.72	2.89 ± 0.81	< 0.001
GLU, median (min-max), mg/dL	133.25 (73.00-45576.82)	141.84 (48.00-111191.78)	0.013
CK, median (min-max), U/L	177.00 (12.00-267540.00)	1040.50 (62.00-127750.00)	< 0.001
CK-MB, median (min-max), U/L	4.00 (1.00-469.00)	16.00 (1.00-548.00)	< 0.001
LDH, median (min-max), U/L	315.00 (91.00-6890.00)	803.00 (147.00-16470.00)	< 0.001
Diagnosis, *n* (%)			0.102
Acute and subacute hepatic failure	551 (90.18%)	272 (93.47%)	
Chronic hepatic failure	60 (9.82%)	19 (6.53%)	
Race, *n* (%)			< 0.001
Asian	32 (5.24%)	6 (2.06%)	
Black	66 (10.80%)	17 (5.84%)	
White	370 (60.56%)	142 (48.80%)	
Other	57 (9.33%)	24 (8.25%)	
Unknown	86 (14.08%)	102 (35.05%)	

### The LAR is an independent risk factor for all-cause mortality in patients with liver failure

Covariates exhibiting significant differences (*P* < 0.05) in Table 1 were included in the univariate Cox regression analysis. The results showed that the unadjusted LAR was significantly associated with all-cause mortality in hepatic failure patients within hospital admission (HR, 1.67; 95% CI, 1.50–1.86; *P* < 0.0001), summarized in Table 2. Covariates with *P* < 0.05 in Table 2 and potential risk factors were then incorporated into multivariate Cox regression analyses. Multivariate Cox proportional hazards regression models were used to assess the association between LAR and all-cause mortality in patients with hepatic failure, are detailed in Table 3. In the adjusted model I, LAR was significantly associated with in-hospital all-cause mortality in patients with hepatic failure (HR, 1.67; 95% CI, 1.50–1.86; *P* < 0.0001) after adjusting for age and weight. Furthermore, after adjusting for age, weight, MELD, SOFA, HR, SBP, DBP, MBP, RR, T, SPO2, MCV, MCHC, MCH, PLT, WBC, ALT, AST, TBIL, INR, PT, and ALP in the fully adjusted model II, LAR remained an independent predictor (HR, 1.66; 95% CI, 1.35–2.05; *P* < 0.0001). These adjustments help to control for potential confounding factors and make the results more reliable in reflecting the true relationship between LAR and all-cause mortality. Therefore, LAR was identified as an independent correlate of the all-cause mortality rate in hepatic failure patients.

**Table 2:** Univariate COX analysis of variables for all-cause motility by logistic regression analysis

Variables	All-cause motility		
	HR	95% CI	*P*-value
LAR	1.67	1.50, 1.86	< 0.0001
Age	1.01	1.00, 1.02	0.0134
Weight	1.01	1.00, 1.01	0.0474
MELD	1.07	1.05, 1.09	< 0.0001
SOFA	1.17	1.12, 1.21	< 0.0001
SBP	0.97	0.96, 0.98	< 0.0001
DBP	0.97	0.96, 0.99	< 0.0001
MBP	0.97	0.96, 0.98	< 0.0001
HR	1.02	1.01, 1.03	< 0.0001
RR	1.14	1.10, 1.17	< 0.0001
T	0.56	0.46, 0.69	< 0.0001
SPO_2_	0.86	0.81, 0.90	< 0.0001
MCH	1.10	1.05, 1.16	< 0.0001
MCHC	1.14	1.05, 1.25	0.0035
MCV	1.03	1.01, 1.05	0.0018
PLT	1.00	1.00, 1.00	0.0001
GLU	1.00	1.00, 1.00	0.335
Lactate	1.18	1.13, 1.22	< 0.0001
Albumin	0.41	0.34, 0.50	< 0.0001
ALT	1.00	1.00, 1.00	0.0116
AST	1.00	1.00, 1.00	< 0.0001
ALP	1.00	1.00, 1.00	0.0131
TBIL	1.08	1.05, 1.10	< 0.0001
PT	1.02	1.01, 1.03	0.0018
INR	1.15	1.02, 1.29	0.0177
CK-MB	1.01	1.00, 1.02	0.024
LDH	1.00	1.00, 1.00	< 0.0001
Race			
Asian	1.00	-	-
Black	1.37	0.49, 3.82	0.5425
White	2.05	0.84, 5.00	0.116
Other	2.25	0.83, 6.07	0.1106
Unknown	6.33	2.53, 15.84	< 0.0001

**Table 3:** Multivariate COX analysis of risk factors for all-cause mortality by logistic regression analysis

Exposure	Non-adjusted		Adjust I		Adjust II	
	HR (95% CI)	*P*-value	HR (95% CI)	*P*-value	HR (95% CI)	*P*-value
LAR	1.67 (1.50, 1.86)	< 0.0001	1.67 (1.50, 1.86)	< 0.0001	1.66 (1.35, 2.05)	< 0.0001
LAR Tertile						
Low	Reference		Reference		Reference	
Middle	2.49 (1.64, 3.79)	< 0.0001	2.46 (1.61, 3.76)	< 0.0001	1.61 (0.76, 3.41)	0.2105
High	8.07 (5.39, 2.08)	< 0.0001	7.87 (5.23, 11.85)	< 0.0001	5.23(2.48, 1.03)	< 0.0001

Non-adjusted model adjusted for: None;

Adjust I model adjusted for: Age and Weight;

Adjust II model adjusted for: Age, Weight, MELD, SOFA, HR, SBP, DBP, MBP, RR, T, SPO_2_, MCV, MCHC, MCH, PLT, WBC, ALT, AST, TBIL, INR, PT, and ALP

### ROC curve analysis and Kaplan–Meier curve

**Fig. 2:**
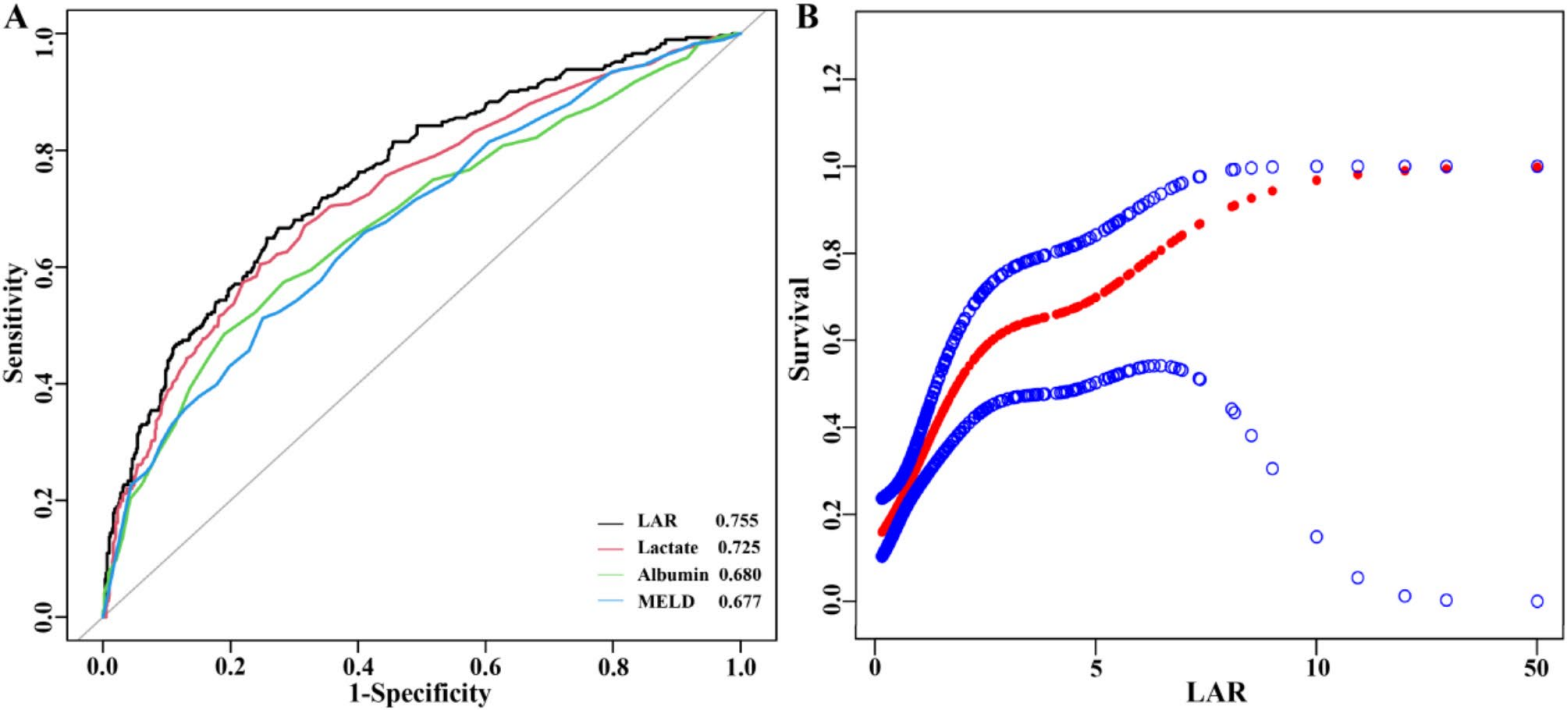
Association between LAR and all-cause mortality of patients with hepatic failure. (**A**) The non-linear relationship between LAR and all-cause mortality. The solid red line represents the smooth curve fit between variables. Blue bands represent the 95% CI from the fit. (**B**) ROC curves assess the predictive capability of the LAR index for all-cause mortality

**Fig. 3:**
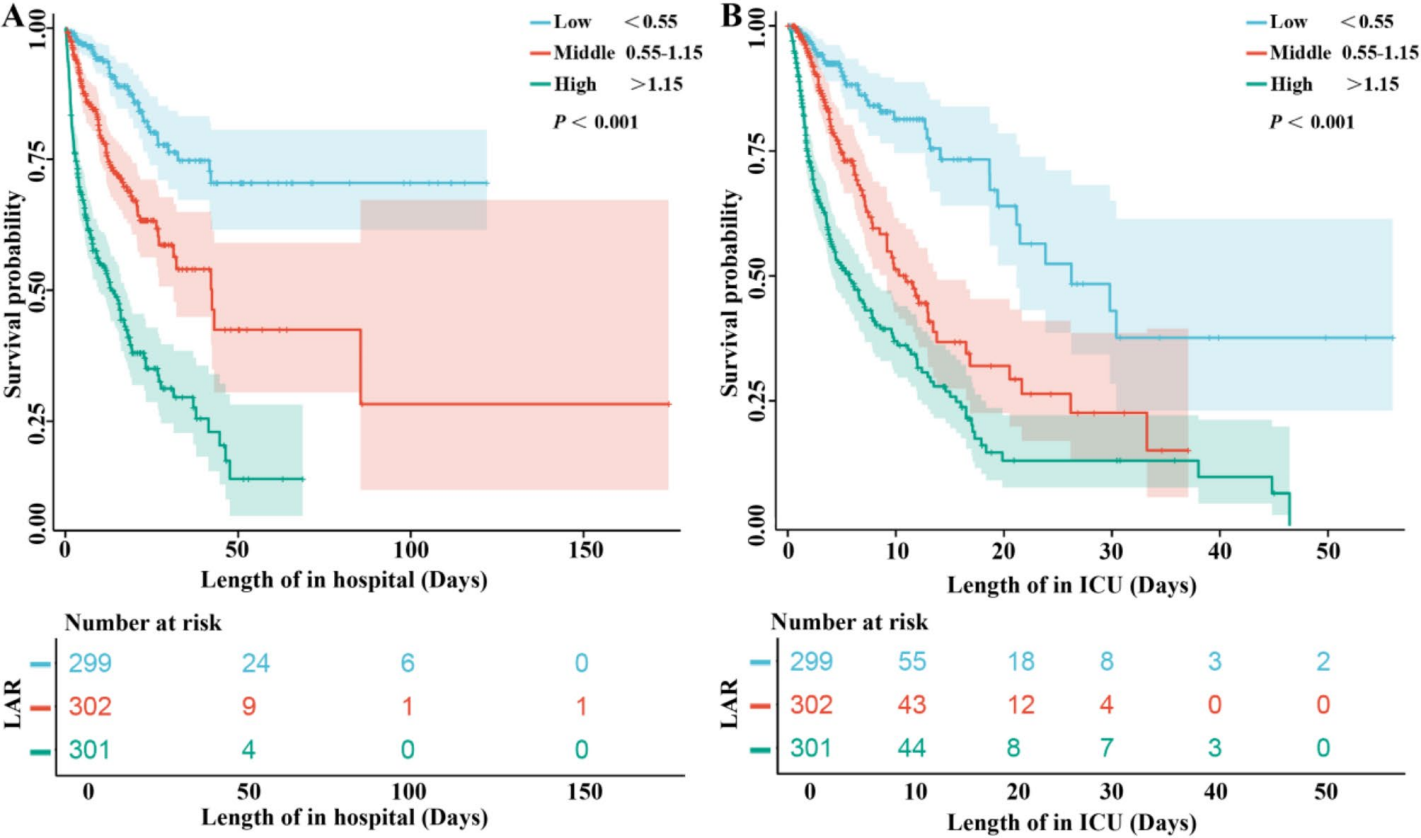
Kaplan-Meier survival analysis curves for all-cause mortality within in-hospital and ICU admission classified into three group according to LAR. (**A**) In-hospital admission. (**B**) ICU admission

We plotted ROC curves for the four indicators of LAR, LAC, ALB, and MELD to predict all-cause mortality in hepatic failure patients within hospital admission, as depicted in Fig. 2A and summarized in Table 4. The AUC of LAR [0.755 (0.721, 0.789)] notably outperformed LAC [0.725 (0.689, 0.762)] and ALB [0.680 (0.641, 0.718)]. Notably, when compared with LAR, MELD did demonstrate a significant inferiority [0.677 (0.639, 0.714)]. The results presented in Supplementary Table 1, revealed that all the *P* < 0.001, indicating a highly statistically significant difference among the AUC of these markers. Moreover, we determined the optimal cut-off value for LAR to be 0.97, with sensitivity of 65.0% and specificity of 74.30%, indicating that LAR had a relatively good predictive ability for all-cause mortality in patients with hepatic failure. With LAR categorized into low (LAR < 0.55, *n* = 299), middle (0.55 ≤ LAR ≤ 1.15, *n* = 302), and high groups (LAR > 1.15, *n* = 301) based on tertiles, Kaplan-Meier survival analysis curves were plotted (Fig. 3), which showed statistically significant difference between the all-cause mortality rate of the three groups (*P* < 0.001). The mortality rates of patients in the low LAR group were significantly lower than those in the middle and high LAR groups. Patients with elevated LAR showed a significantly heightened risk of in-hospital mortality, further demonstrating the prognostic value of LAR.

**Table 4:** Information of ROC curves in Fig. 2A

Test	Non-survivors	Survivors	AUC (95%CI)	Best threshold	Specificity	Sensitivity	Youden’s index
LAR	291	611	0.755 (0.721, 0.789)	0.971	0.743	0.650	0.393
Lactate	291	611	0.725 (0.689, 0.762)	3.150	0.753	0.605	0.358
Albumin	291	611	0.680 (0.641, 0.718)	2.750	0.810	0.485	0.295
MELD	291	611	0.677 (0.639, 0.714)	27.500	0.750	0.512	0.262

### The analysis of the non-linear association

To detect the potentially non-linear relationship between the LAR and the risk of all-cause mortality in patients with hepatic failure, a smooth curve fitting approach and threshold effect analysis were employed. Two models were developed, each adjusted for different sets of covariates to enhance the accuracy of the results. The Adjust I model accounted for age and weight, while the Adjust II model incorporated a more comprehensive set of variables. After controlling for potential confounding factors, a significant non-linear correlation emerged between LAR and hepatic failure (*P* for non-linearity < 0.001), are presented in Fig. 2B. This finding indicates that the relationship between LAR and the outcome is more complex than a simple linear association. The threshold effect analysis further revealed a crucial inflection point of 1.33 for LAR (Table 5). The *P*-value for the log-likelihood ratio test was 0.015, suggesting a better fit of the two-piecewise linear regression model.

**Table 5:** Information of nonlinear associations between LAR and all-cause mortality in Fig. 2B

Outcome	All-cause mortality	
	HR (95% CI)	*P*-value
Model I: Fitting model by standard linear regression	1.66 (1.31, 2.23)	< 0.0001
Model II: Fitting model by two-piecewise linear regression		
Inflection point	1.33	
≤ 1.33	4.86 (1.98, 11.95)	0.0006
> 1.33	1.31 (1.00, 1.70)	0.0472
*P*-value for log likelihood ratio test		0.015

Adjust I model adjusted for: Age and Weight;

Adjust II model adjusted for: Age, Weight, MELD, SOFA, HR, SBP, DBP, MBP, RR, T, SPO_2_, MCV, MCHC, MCH, PLT, WBC, ALT, AST, TBIL, INR, PT, and ALP

### Subgroup analysis

According to the LAR cutoff value of the K-M survival curves, the patients were divided into three groups: low, middle, and high. Table 6 compared the baseline characteristics in the three groups. As the LAR increased, all-cause mortality in hospital with liver failure increases gradually. Besides, so did age, MELD, SOFA, HR, RR, WBC, MCV, RDW, LAC, ALT, AST, TBIL, DBIL, IBIL, PT, INR, CK, CK-MB, and LDH. SBP, DBP, MBP, T, SPO_2_, PLT, and ALB decreased as the LAR increased. Table 7 shows the results of subgroup analysis based on liver failure classification. In both acute and subacute hepatic failure and chronic hepatic failure groups, LAR was significantly higher in non-survivors compared to survivors. The median LAR in non-survivors with acute and subacute hepatic failure was 1.57 (0.18-35.00), and in those with chronic hepatic failure was 1.06 (0.52–8.14), indicating that LAR could also predict mortality in different types of liver failure.

**Table 6:** Baseline characteristics of the population according to LAR

Variables	LAR			*P*-value
	Low (< 0.55)	Middle (0.55–1.15)	High (> 1.15)	
N	299	302	301	
All-cause mortality, *n* (%)				< 0.001
Survivors	259 (86.62%)	218 (72.19%)	134 (44.52%)	
Non-survivors	40 (13.38%)	84 (27.81%)	167 (55.48%)	
Gender				0.239
Female	127 (42.47%)	109 (36.09%)	124 (41.20%)	
Male	172 (57.53%)	193 (63.91%)	177 (58.80%)	
Age, mean ± SD, year	57.38 ± 16.00	61.00 ± 15.17	61.34 ± 15.59	< 0.001
Height, mean ± SD, cm	169.52 ± 10.22	169.71 ± 10.88	168.59 ± 10.26	0.458
Weight, mean ± SD, kg	83.82 ± 20.67	86.16 ± 24.22	85.70 ± 25.89	0.450
MELD, mean ± SD	21.28 ± 9.09	22.33 ± 9.40	25.67 ± 9.93	< 0.001
SOFA, mean ± SD	8.61 ± 4.39	9.42 ± 3.89	11.59 ± 4.09	< 0.001
LAR, mean ± SD	0.38 ± 0.10	0.77 ± 0.17	3.45 ± 3.40	< 0.001
SBP, mean ± SD, mmHg	112.97 ± 15.04	111.15 ± 13.65	108.78 ± 15.44	0.002
DBP, mean ± SD, mmHg	63.38 ± 10.90	62.26 ± 11.02	60.93 ± 11.34	0.026
MBP, mean ± SD, mmHg	77.69 ± 10.39	76.25 ± 10.17	74.97 ± 10.96	0.007
HR, mean ± SD, beats/min	88.48 ± 16.85	87.54 ± 16.96	93.00 ± 18.66	< 0.001
RR, mean ± SD, breath/min	20.19 ± 4.45	20.07 ± 4.31	22.10 ± 4.98	< 0.001
T, mean ± SD, ℃	36.85 ± 0.65	36.84 ± 0.59	36.61 ± 1.00	< 0.001
SPO_2_, mean ± SD, %	96.75 ± 2.56	96.50 ± 2.66	95.79 ± 4.59	0.001
WBC, median (min-max), 10^9^/L	8.10 (0.50–36.50)	8.75 (1.50–72.30)	11.50 (0.90–77.90)	< 0.001
RBC, mean ± SD, 10^12^/L	3.40 ± 0.76	3.28 ± 0.86	3.29 ± 0.81	0.162
HCT, median (min-max), %	31.02 ± 6.39	30.05 ± 7.14	30.66 ± 7.08	0.220
Hb, mean ± SD, mg/dL	10.08 ± 2.18	9.75 ± 2.37	9.98 ± 2.32	0.193
MCH, mean ± SD, pg	29.93 ± 3.03	30.11 ± 3.40	30.53 ± 2.77	0.056
MCHC, mean ± SD, g/L	32.46 ± 1.61	32.45 ± 1.59	32.55 ± 1.63	0.731
MCV, mean ± SD, fl.	92.23 ± 7.56	92.75 ± 9.03	93.96 ± 8.16	0.037
RDW, mean ± SD, %	16.35 ± 3.09	17.33 ± 3.42	17.33 ± 3.42	0.002
PLT, median (min-max), 10^9^/L	161.00 (15.00-606.00)	144.00 (15.00-603.00)	135.50 (12.00-773.00)	0.006
Lactate, median (min-max), mmol/L	1.35 ± 0.40	2.41 ± 0.71	8.92 ± 6.63	< 0.001
Albumin, mean ± SD, g/mL	3.65 ± 0.67	3.15 ± 0.70	2.89 ± 0.78	< 0.001
ALT, median (min-max), U/L	55.00 (6.00-5372.00)	52.00 (8.00-6342.00)	120.00 (5.00-8648.00)	< 0.001
AST, median (min-max), U/L	57.00 (6.00-8965.00)	83.00 (5.00-15960.00)	173.00 (13.00-42606.00)	< 0.001
ALP, median (min-max), U/L	109.00 (32.00-1020.00)	119.50 (40.00-1199.00)	122.00 (22.00-2686.00)	0.320
TBIL, median (min-max), mg/dL	0.90 (0.20–42.20)	1.40 (0.20–52.20)	1.70 (0.20–39.30)	< 0.001
DBIL, median (min-max), mg/dL	1.00 (0.10–24.00)	2.00 (0.40–21.60)	4.05 (0.30–18.20)	0.042
IBIL, median (min-max), mg/dL	0.70 (0.20–9.60)	2.00 (0.20–11.10)	1.30 (0.20–11.30)	0.016
GLU, median (min-max), mg/dL	134.08 (74.50-448.50)	134.25 (73.00-111191.78)	142.58 (48.00-547.83)	0.170
PT, median (min-max), s	14.70 (9.40–150.00)	16.05 (9.50–89.30)	18.20 (10.30-115.40)	< 0.001
INR, median (min-max)	1.40 (0.90–13.80)	1.50 (0.90–15.70)	1.70 (0.90–10.30)	< 0.001
CK, median (min-max), U/L	166.00 (21.00-17911.00)	243.50 (12.00-139580.00)	1063.50 (13.00-267540.00)	< 0.001
CK-MB, median (min-max), U/L	5.00 (1.00-469.00)	5.00 (1.00-206.00)	16.00 (1.00-548.00)	< 0.001
LDH, median (min-max), U/L	313.00 (91.00-5440.00)	353.50 (104.00-9290.00)	695.00 (134.00-16470.00)	< 0.001
Diagnosis, *n* (%)				0.007
Acute and subacute hepatic failure	272 (90.97%)	265 (87.75%)	286 (95.02%)	
Chronic hepatic failure	27 (9.03%)	37 (12.25%)	15 (4.98%)	
Race, *n* (%)				< 0.001
Asian	15 (5.02%)	12 (3.97%)	11 (3.65%)	
Black	31 (10.37%)	26 (8.61%)	26 (8.64%)	
White	186 (62.21%)	181 (59.93%)	145 (48.17%)	
Other	29 (9.70%)	26 (8.61%)	26 (8.64%)	
Unknown	38 (12.71%)	57 (18.87%)	93 (30.90%)	

**Table 7:** Subgroup analysis of liver failure classification

	All-cause mortality		*P*-value
	Survivors	Non-survivors	
Total	611	291	
LAR [median (min-max)]	0.61 (0.14–23.42)	1.46 (0.18-35.00)	< 0.001
Acute and subacute hepatic failure	551	272	
LAR [median (min-max)]	0.61 (0.15–23.42)	1.57 (0.18-35.00)	< 0.001
Chronic hepatic failure	60	19	
LAR [median (min-max)]	0.57 (0.14–5.45)	1.06 (0.52–8.14)	0.004

## Discussion

Based on 902 hepatic failure patients in the MIMIC-IV database, this large retrospective study found that the LAR was an independent factor for all-cause mortality in hepatic failure patients at hospitalization after adjustment for potential confounding factors. The comparison of the AUC values indicated that LAR demonstrates a higher accuracy compared to lactate or albumin alone. Furthermore, it even outperforms MELD. Meanwhile, Kaplan-Meier survival analysis plots indicate that patients with LAR > 1.15 had significantly higher all-cause mortality within hospitalization than those with LAR < 0.55. Additionally, two-piecewise linear regression model showed the results to be generally robust. The results of subgroup analysis showed acute or subacute hepatic failure and chronic hepatic failure groups, LAR was significantly higher in non-survivors compared to survivors. Such results contribute to a more comprehensive understanding of the role of LAR in evaluating the prognosis of hepatic failure patients, potentially providing valuable insights for clinical decision-making and risk assessment.

Liver failure is a clinical syndrome characterized by severe hepatic dysfunction leading to multiorgan failure in patients with end-stage liver disease [20, 21]. Liver failure is a challenging clinical syndrome with a rapid clinical course and high short-term mortality [5, 20]. In recent years, researchers have been increasingly focused on developing scoring models to predict the prognosis of patients with liver failure. Several scoring systems have been developed and utilized in the management of liver failure, aiming to improve risk stratification, optimize resource allocation, and enhance patient care. These models include well-known systems such as the MELD, CTP, King’s College Hospital (KCH), Chronic Liver Failure Consortium-Organ failure scores (CLIF-C OFs), Chronic Liver Failure Consortium-Acute-on-Chronic liver failure (CLIF-C ACLFs), Chronic Liver Failure-Sequential organ failure assessment (CLIF-SOFA), and Chinese Group on the Study of Severe Hepatitis-Acute-on-Chronic liver failure (COSSH-ACLF) [2–6]. In addition to these scoring systems, metabolic disorder markers [22–24], inflammation markers [25–27], and oxidative stress markers [28, 29] have been identified as potential indicators for providing early warnings and accurate prognosis predictions before the onset of organ failure, which play a crucial role in improving the clinical prognosis of patients with liver failure. However, there is still no consensus in predicting liver failure-related outcomes, which makes comparing studies difficult and standardizing management protocols challenging. One promising marker that has garnered attention is the LAR. While LAR has been successfully applied as a predictor in various conditions such as sepsis, acute pancreatitis, heart failure, and acute respiratory failure [13, 30–32], its utility in predicting the prognosis of liver failure patients has not yet been studied. Further research exploring the application of LAR in predicting the prognosis of patients with liver failure could potentially provide valuable insights and improve clinical outcomes for this patient population.

Lactate, a metabolic byproduct, serves as a vital component in evaluating critically ill patients. Our study demonstrated that hyperlactatemia correlates with an unfavorable prognosis in patients with liver failure, aligning with previous research findings [33]. The liver, as a central metabolic organ, plays a pivotal role in lactate metabolism, chiefly through the Cori cycle and gluconeogenesis. In liver failure, this metabolic pathway becomes compromised, resulting in decreased lactate clearance. Consequently, serum lactate levels elevate, indicating impaired liver function and concurrent tissue hypoxia, commonly observed in critically ill patients with liver failure. Yi et al. ascertained that an elevated lactate concentration served as an independent risk factor for 28-day mortality among individuals afflicted with sepsis-associated liver injury [14]. In a similar vein, Ma et al. demonstrated that LAR exhibited a positive correlation with disease severity and an unfavorable short-term prognosis in patients suffering from COSSH ACLF [34]. Hence, heightened lactate levels in liver failure are linked to a dismal prognosis and heightened mortality rates [7, 8, 10, 35]. Albumin, a crucial protein synthesized by the liver, is integral to various physiological processes [36]. Its levels have been identified as a prognostic risk factor in multiple medical conditions. For instance, in cervical cancer patients, serum albumin levels were found to be a significant risk factor for prognosis [37]. Similarly, in elderly patients with sepsis or COVID-19, albumin emerged as an independent predictor of mortality in a cohort study, indicating higher mortality rates with decreasing albumin levels [38, 39]. Albumin’s significance extends beyond its role as a marker. It possesses essential oncotic and transport functions, along with immunomodulatory and antioxidant properties, all of which are pertinent in the context of liver failure. Despite its simplicity and widespread availability, elevated lactate levels may also signify systemic inflammatory response syndrome and sepsis. serum albumin levels can increase following the infusion of human albumin, particularly when left untreated. Therefore, each factor alone may not fully capture the complex dynamics of liver failure progression. We propose a novel approach to enhance the accuracy of predicting the prognosis of liver failure patients by introducing a ratio of blood lactate to serum albumin. This innovative method aims to mitigate the impact of a single factor on the regulatory mechanism by considering the inverse changes induced by two distinct physiological pathways [40].

In liver disease, LAR is intimately tied to an augmented risk of all-cause mortality within the initial 28 days of patient admission. This discovery strongly intimates that LAR could potentially function as an independent risk factor for unfavorable outcomes in patients afflicted with sepsis-associated liver injury [14]. Delving deeper into the analysis, when the LAR was set at a cutoff value of 0.9061, it exhibited a sensitivity of 66.7% and a specificity of 76.2%, thereby signifying that an elevated LAR holds predictive prowess for in-hospital mortality, particularly in hospitalized cirrhotic patients [41]. When contrasting patients with an LAR of less than 1.01 to those with an LAR equal to or greater than 1.01, it became evident that the latter group endured a notably poorer 28-day prognosis. Consequently, it was firmly established that LAR bore a positive correlation with both disease severity and an unfavorable short-term prognosis in patients grappling with COSSH ACLF [34]. Moreover, while benchmarking the predictive capabilities of various metrics, it was observed that although the predictive value of LAR for the prognosis of patients with cirrhosis combined with sepsis was somewhat less than that of the highly regarded MELD, it nonetheless surpassed, by a significant margin, the predictive powers of lactate, albumin, and the Sequential Organ Failure Assessment. In sum, LAR has demonstrated good predictive value for the prognosis of patients with cirrhosis and sepsis [42]. Additionally, LAR was identified as an early prognostic marker for both 14-day and 28-day mortality in patients diagnosed with urosepsis [43, 44]. However, while these findings enhance our understanding of the prognostic implications of LAR in various diseases, they do not directly address its role in liver failure. Our study delved into separate multivariable regression analyses for LAR, lactate, and albumin. We observed that patients with liver failure exhibited a higher risk of mortality with increasing LAR levels. Moreover, Kaplan-Meier survival curve analysis demonstrated mortality rates of patients in the low LAR group were significantly lower than those in the middle and high LAR groups. We discovered a non-linear association between the LAR and the risk of hospital mortality after adjusting for potential confounders. These findings offer valuable insights into prognostic indicators for liver failure, particularly emphasizing the association between LAR and adverse outcomes in patients with liver failure. Further research in this area could provide additional clarity and potentially inform clinical management strategies for these patients.

We must acknowledge some limitations of this study. Firstly, the precise etiology of liver failure progression in all patients was not traceable in our study due to the limitations of the MIMIC-IV database. The majority of patients in this study were diagnosed with acute and subacute hepatic failure (approximately 91.37%). However, in the USA and Western Europe, drug-induced liver injury is the most common cause of acute liver failure, while in developing countries, viral hepatitis remains predominant. Therefore, it remains uncertain whether these conclusions are applicable to other countries and races. Rigorous prospective randomized controlled trials are still needed to confirm the findings in the future. Secondly, our study is a single-center retrospective cohort study, and the relationship between LAR and liver failure prognosis was measured for the first time after hospital admission. Liver failure is a dynamic process, and the best prognostic markers are those that reflect the clinical evolution of organ failure over time rather than assessment at a single time point. This limitation restricts our ability to assess the prognostic impact of dynamic LAR, thus diminishing the persuasive power of our findings. Finally, our dataset may include patients with comorbidities such as renal disease and diabetes, the medication regimen before blood samples were collected for lactate and albumin determination was unclear, which could potentially affect lactate and albumin levels. Further subgroup analysis is warranted to establish the robustness of our results.

## Conclusion

In our study, LAR emerged as an independent predictor of all-cause mortality among liver failure patients during hospitalization, and demonstrating surpass to MELD. The association between LAR and in-hospital mortality in critically ill patients displayed a non-linear pattern, with an identified inflection point at 1.33. As LAR levels increase, there is a corresponding elevation in the mortality rate. This finding underscores the importance of monitoring LAR levels in clinical practice, providing healthcare professionals with a valuable tool for early intervention and clinical decision-making regarding patients with poor outcomes. Nevertheless, further validation through large-scale multicenter prospective studies is warranted to establish the widespread applicability and objectivity of LAR as a prognostic indicator in liver failure patients.

## Electronic supplementary material

Below is the link to the electronic supplementary material.

Supplementary Material 1

## Data Availability

Data associated with this study has been deposited at the data are available on the MIMIC-IV database website at https://mimic-iv.mit.edu/ and the GitHub website at https://github.com/MIT-LCP/mimic-code.

## Declarations

### Abbreviations

All abbreviations used in this study can be found in Supplementary Table 2.

### Foundation

This work was supported by the Science and Technology Fund Project of Guizhou Health Commission in 2024 (No. gzwkj2024-010), the Guizhou Provincial Science and Technology Projects (QKH JC-ZK [2024]-210; QKH JC-zk[2025]-461), and Cultivation Program for Regional Projects of the National Natural Science Foundation of China (No. gyfynsfc[2024]-19).
