# Supplementary material for: Association between lactate-to-albumin ratio and mortality in hepatic failure: a retrospective cohort study
Source: BMC Infect Dis. 2025 Mar 28;25:433. doi: 10.1186/s12879-025-10783-z (PMC11951681; doi:10.1186/s12879-025-10783-z)
Supplement: Supplementary file 1 — Supplementary Material 1 [file 12879_2025_10783_MOESM1_ESM.pdf]

Supplementary material Table 1. Delong's test results

Test	AUC	Standard Error	*P*-value	95% CI	
				Lower	Upper
LAR	0.755	0.017	2.629×10^{-35}	0.211	0.279
Lactate	0.725	0.018	6.369×10^{-28}	0.239	0.311
Albumin	0.680	0.020	2.613×10^{-18}	0.641	0.718
MELD	0.677	0.019	8.574×10^{-18}	0.286	0.361

Supplementary material Table 2. List of abbreviations

Abbreviation	
MIMIC	Medical Information Mart for Intensive Care
LAR	lactate/albumin ratio
ROC	receiver operating characteristic
ICU	intensive care unit
AUC	area under the curve
MELD	Model for End-stage Liver Disease
CTP	Child-Turcotte-Pugh
AARC	APASL ACLF Research Consortium
ABIC	Age-Bilirubin-INR-Creatinine
MDF	Maddrey Discriminant Function
MESO	Model to Estimate Survival in Overt Hepatic Failure
ALBI	Albumin-Bilirubin
APACHE	Acute Physiology and Chronic Health Evaluation
CLIF-C	Chronic Liver Failure Consortium
KCH	King’s College Hospital
CLIF-C OFs	Chronic Liver Failure Consortium-Organ failure scores
CLIF-C ACLFs	Chronic Liver Failure Consortium-Acute-on-Chronic liver failure
CLIF-SOFA	Chronic Liver Failure-Sequential organ failure assessment
COSSH-ACLF	Chinese Group on the Study of Severe Hepatitis-Acute-on-Chronic liver failure
IQR	inter quartile ranges
CI	confidence interval
HR	hazard ratio
OR	odds ratio
SBP	systolic blood pressure
DBP	diastolic blood pressure
MBP	mean blood pressure
SOFA	sequential organ failure assessment
WBC	white blood cell
RBC	red blood cell
HCT	Hematocrit
MCH	mean corpuscular hemoglobin
MCHC	mean corpuscular hemoglobin concentration
RDW	red blood cell distribution width
PLT	platelet
ALT	alanine aminotransferase
AST	aspartate aminotransferase
TBIL	total bilirubin
DBIL	direct bilirubin
IBIL	indirect bilirubin
ALB	albumin

ALP	alkaline phosphatase
INR	international normalized ratio
PT	prothrombin time
LAC	lactate
GLU	glucose
LDH	lactate dehydrogenase
CK	ceatine kinase
CK-MB	creatine kinase isoenzyme
T	temperature
HR	heart rate
RR	respiratory rate
SPO2	oxygen saturation
SD	standard deviation
SE	standard error
